# The renal blood flow reserve in healthy humans and patients with atherosclerotic renovascular disease measured by positron emission tomography using [^15^O]H_2_O

**DOI:** 10.1186/s13550-018-0395-3

**Published:** 2018-06-11

**Authors:** Johanna Päivärinta, Niina Koivuviita, Vesa Oikonen, Hidehiro Iida, Kaisa Liukko, Ilkka Manner, Eliisa Löyttyniemi, Pirjo Nuutila, Kaj Metsärinne

**Affiliations:** 1 Department of Nephrology, Division of Medicine, Turku University Hospital, PL 52, Kiinamyllynkatu 4-8, 20521 Turku, Finland; 2 Turku PET Centre, University of Turku, Turku, Finland; 3 Department of Radiology, Turku University Hospital, Turku, Finland; 4 Department of Biostatistics, University of Turku, Turku, Finland; 5 Department of Medicine, University of Turku, Turku, Finland

**Keywords:** Microvasculature, Renal artery stenosis, Renal flow reserve, Positron emission tomography, Kidney impairment

## Abstract

**Background:**

Microvascular function plays an important role in ARVD (atherosclerotic renovascular disease). RFR (renal flow reserve), the capacity of renal vasculature to dilate, is known to reflect renal microvascular function. In this pilot study, we assessed PET (positron emission tomography)-based RFR values of healthy persons and renal artery stenosis patients.

Seventeen patients with ARVD and eight healthy subjects were included in the study. Intravenous enalapril 1 mg was used as a vasodilatant, and the maximum response (blood pressure and RFR) to it was measured at 40 min. Renal perfusion was measured by means of oxygen-15-labeled water PET. RFR was calculated as a difference of stress flow and basal flow and was expressed as percent [(stress blood flow − basal blood flow)/basal blood flow] × 100%.

**Results:**

RFR of the healthy was 22%. RFR of the stenosed kidneys of bilateral stenosis patients (27%) was higher than that of the stenosed kidneys of unilateral stenosis patients (15%). RFR of the contralateral kidneys of unilateral stenosis patients was 21%. There was no difference of statistical significance between RFR values of ARVD subgroups or between ARVD subgroups and the healthy. In the stenosed kidneys of unilateral ARVD patients, stenosis grade of the renal artery correlated negatively with basal (*p* = 0.04) and stress flow (*p* = 0.02). Dispersion of RFR values was high.

**Conclusions:**

This study is the first to report [^15^O]H_2_O PET-based RFR values of healthy subjects and ARVD patients in humans. The difference between RFR values of ARVD patients and the healthy did not reach statistical significance perhaps because of high dispersion of RFR values. [^15^O]H_2_O PET is a valuable non-invasive and quantitative method to evaluate renal blood flow though high dispersion makes imaging challenging. Larger studies are needed to get more information about [^15^O]H_2_O PET method in evaluation of renal blood flow.

## Background

Renal microvasculature and endothelial function are pivotal in acute and CKD (chronic kidney diseases). In CKD, there are many endothelium-dependent abnormalities like decreased vasodilatation response; decreased amount of angiogenic factors; and increased amount of oxidative stress, inflammation, and capillary permeability [1]. The loss of glomerular and peritubular capillaries has been described in animal studies of CKD [2, 3].

In ARVD (atherosclerotic renovascular disease) patients, there is a combination of renal micro- and macrovascular disease. Three large prospective trials failed to show advantage of renal artery stenosis dilatation [4–6] which was probably due to renal microvascular dysfunction. Iliescu et al. have reported that after intrarenal delivery of vascular endothelial growth factor in animals, renal blood flow and GFR normalize in the stenosed kidneys [7]. Furthermore, renal cortical perfusion has shown to increase in humans after autologous mesenchymal stem cell transplantation in renovascular disease [8].

There has been high hopes that RFR (renal flow reserve), the difference between stress and basal flow, would be a good marker of microvascular function as CFR (coronary flow reserve) which is a strong predictor of future cardiovascular events [9]. CFR is methodologically equivalent to RFR; however, RFR is known to be smaller than CFR [10].

RFR has been studied with different techniques. PET (positron emission tomography) seems to have two remarkable advantages compared to other methods: it is non-invasive and quantitative. With PET, it is also possible to measure regional single-kidney perfusion without contrast agent [11–14]. Many different vasostimulants have been used in the studies of RFR, too. ACE inhibitor-induced vascular response in ARVD patients has been evaluated in several animal studies with different methods. However, human studies are mainly based on renography [15, 16].

In this study, we evaluated RFR values of the healthy and ARVD patients by [^15^O]H_2_O PET using enalapril as a vasostimulant. We have previously reported basal flow values of the same study population [11]. This is a pilot study of PET-based renal flow reserve values induced by enalapril in ARVD patients and the healthy.

## Methods

### Subjects

Seventeen patients with ARVD and eight healthy control subjects were included in the study. The patients were recruited from the nephrological outpatient clinic of Turku University Hospital. ARVD was defined as a stenosis of > 60% of the renal artery as determined by digital subtraction angiography.

### Study design

Each patient was studied twice, once before the dilatation of RAS (renal artery stenosis) and the second time 103+/−29 days after revascularization. The imaging studies were performed after a 10-h overnight fast. All patients were instructed to interrupt their antihypertensive medication on the study day and ACEI (angiotensin converting enzyme inhibitor) or ARB (angiotensin receptor blocker) medication 3 days before the study day.

Alcohol, smoking, and caffeine were prohibited for 3 days before assessment. Subjects with symptoms of acute infections within a week prior to or during the study were excluded from the analysis.

A venous catheter was inserted into an antecubital vein for injecting [^15^O]H_2_O. After the first scan, the ARVD patients were given 1 mg enalapril-infusion in 5 min. Scans were taken at 20, 40, and 60 min after enalapril-infusion. The flow was at maximum at 40 min after enalapril and that value was chosen to analysis. The examinees were supposed to be immobile between the scans.

### Image acquisition, processing, and correction

Renal perfusion was measured with GE advance PET tomography (General Electric, Milwaukee, Wisconsin) as previously described [17]. PET data were corrected for dead time, decay, and measured photon attenuation. Images were processed with the standard reconstruction algorithm (standard = the ordered-subsets expectation-maximization method using a Hann filter with a cutoff frequency of 4.6 mm).

### Calculation of RBF and RFR

ROI (regions of interest) for the whole cortical region of the kidneys were drawn on a summed reconstructed image on an average of six coronal planes. For the calculation of renal perfusion from the PET study, the input function was estimated using an average TAC (time activity curve) from descending aorta cavity ROIs [14] drawn on average three planes.

Delay between the renal and aorta TAC was corrected, but due to the large size of the aorta, recovery correction was not considered necessary. Renal perfusion images were generated from the reconstructed dynamic image and the obtained input function by a basis function method assuming a single tissue compartment model [17]. The renal perfusion was represented by the clearance rate (k2) multiplied with the physiological partition coefficient, i.e., p(phys) = 0.94 mL/g [17]. The mean renal perfusion values were obtained from the renal perfusion images using ROIs drawn on summed images.

The ROIs drawn for the cortical region as described above after the first scan were moved as such to the second scan. RFR was calculated as the difference of stress flow and basal flow and was expressed as percent [(stress blood flow − basal blood flow)/basal blood flow] × 100%.

### Statistical analysis

Results are expressed as mean values together with range or standard deviation. Pearson correlations were calculated to study association between numerical variables. For patients having bilateral disease, and for control subjects, mean values of flow for both kidneys were calculated. Some calculations were made including all the diseased kidneys, both from bilateral subjects and the stenosed kidneys from unilateral RAS subjects and also separately for diabetic subjects and non-diabetic subjects.

*P* values less than 0.05 (two-tailed) were considered as statistically significant. The program used for statistical analysis was SAS® System version 9.4 for Windows (SAS Institute Inc., Cary, NC, USA).

## Results

### Baseline and follow-up demographic and clinical data

The characteristics of the study subjects are shown in Table 1.

**Table 1:** Baseline data

	ARVD	Unilateral RAS	Bilateral RAS	Healthy
N	17	8	9	8
Age	69 (52–85)	66 (54–79)	71 (52–85)	60 (48–75)
Male/female	7/10	4/4	3/6	5/3
eGFR (ml/min)	56 (23)	62 (24)	54 (21)	75 (6)
RAS severity (22 kidneys)				
60–80%	15	4	11	0
> 80%	7	3	4	0
Diabetes (all type 2)	9	2	7	0
Coronary heart disease (*n*/%)	6/35	1/13	5/56	0
Peripheral vascular disease (*n*/%)	4/24	1/13	3/33	0
Cerebrovascular disease (*n*/%)	5/29	1/13	4/44	0
Smoking (*n*/%)	4/24	1/13	3/33	0

Values (except age) are expressed as mean (SD)

*N* number of patients, *eGFR* estimated glomerular filtration rate, *RAS* renal artery stenosis, *MAP* mean arterial pressure, [diastolic blood pressure + (systolic blood pressure-diastolic blood pressure)/3]

Coronary heart disease was defined by symptomatic angina, positive exercise test, angiographic evidence of coronary artery disease, or history of previous myocardial infarction. Cerebrovascular disease was defined by medical history, clinical signs, and/or radiologic confirmation of a transient ischemic attack or cerebrovascular accident. Peripheral vascular disease was defined by symptoms of intermittent claudication, previous surgery for lower limb arterial insufficiency, and/or angiographic evidence of significant stenosis in one or more blood vessels that supply lower limbs.

The indication for revascularization in all patients was refractory or treatment-resistant hypertension. Unilateral ARVD patients (*n* = 8): five patients had stenosis in the right renal artery and three patients at the left side. Two of the patients had two renal arteries to the stenosed kidney; the dilated atherosclerotic stenosis was in the larger arteries. All but one patient received a stent during the angioplasty procedure, and the technical outcome was good in every patient (no residual stenosis after dilatation).

Bilateral ARVD patients (*n* = 9): three patients had significant RAS of the dilated side and total occlusion of the contralateral side unsuitable for dilatation. In five patients, both left and right renal arteries were dilated. In one patient, revascularization was done only to the left side, while the stenosis in the right was marginal. Two of the patients had two renal arteries, but the stenosis was in the dominant artery. All patients received at least one stent during the procedure. Unlike in unilateral RAS patients, some degree (</= 25%) of residual stenosis was seen in most of the bilateral RAS patients after dilatation. In one patient, the renal artery was dissected during the stent placement and a surgical bypass operation was done.

All patients were on antihypertensive medication. Fourteen patients used either ACEIs or ARBs, and beta blockers, 13 used calcium channel blockers, and 16 used diuretics. Two patients used a combination of two medications, four patients a combination of three, four patients a combination of four, five patients a combination of five, and two patients a combination of six medications.

The healthy control subjects were normotensive and had normal creatinine, and none of them used any medication.

### RFR values of the healthy control subjects and of ARVD patients

RFR of the healthy subjects was 22 +/− 32%. In the contralateral kidneys of unilateral stenosis patients, RFR was 21 +/− 26%. RFR of the stenosed kidneys of unilateral stenosis patients was 15 +/− 22%, and RFR of bilateral stenosis patients was 27 +/− 43%. In all the stenosed kidneys of diabetics, RFR was 24 +/− 41%, and in all the stenosed kidneys of non-diabetics, RFR was 14 +/− 19%. There was no statistically significant difference in RFR values between any subgroup of ARVD patients or between the healthy and ARVD patients before dilatation (*p* = NS). Diabetes did not have any statistically significant effect on predilatation RFR values either (*p* = NS). RFR and flow values are reported in Table 2.

**Table 2:** RFR and flow values in the healthy and RAS patients

	*N*	Predilatation		
		Basal flow (ml/min/g)	Stress flow (ml/min/g)	RFR (%)
Healthy	8	1.8 (0.3)	2.2 (0.6)	22 (32)
RAS unilateral contralateral kidneys	7	1.8 (0.7)	2.1 (0.6)	21 (27)
RAS unilateral, stenosed kidneys	7	1.5 (0.7)	1.7 (0.7)	15 (22)
RAS bilateral	9	1.4 (0.3)	1.7 (0.4)	27 (43)
Diabetics, stenosed kidneys	13	1.3 (0.4)	1.6 (0.5)	24 (41)
Non-diabetics, stenosed kidneys	9	1.6 (0.5)	1.8 (0.5)	14 (20)

Values are expressed as mean (SD)

*N* number of patients in the subgroups of healthy and bilateral, *RAS* number of the kidneys in other groups, *RFR* renal flow reserve, calculated as [(stress blood flow-basal blood flow)/basal blood flow] × 100%

### Basal and stress flow in ARVD patients

**Fig. 1:**
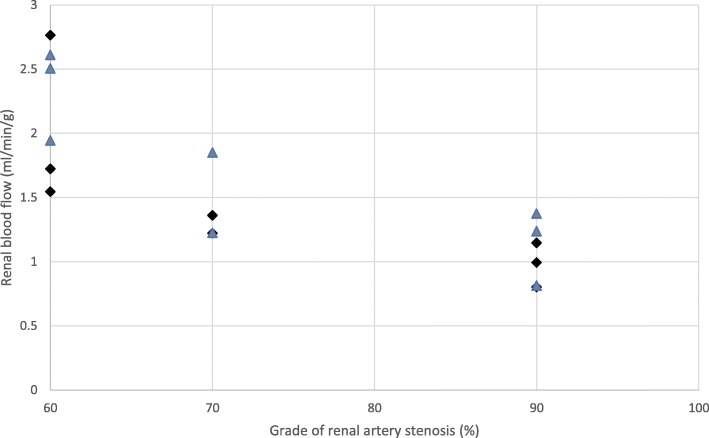
Grade of renal artery stenosis vs renal blood flow in unilateral RAS. ♦Grade of renal artery stenosis vs basal flow in unilateral RAS (*r* = 0.8, *p* = 0.04).  Grade of renal artery stenosis vs stress flow in unilateral RAS (*r* = 0.8, *p* = 0.02)

**Fig. 2:**
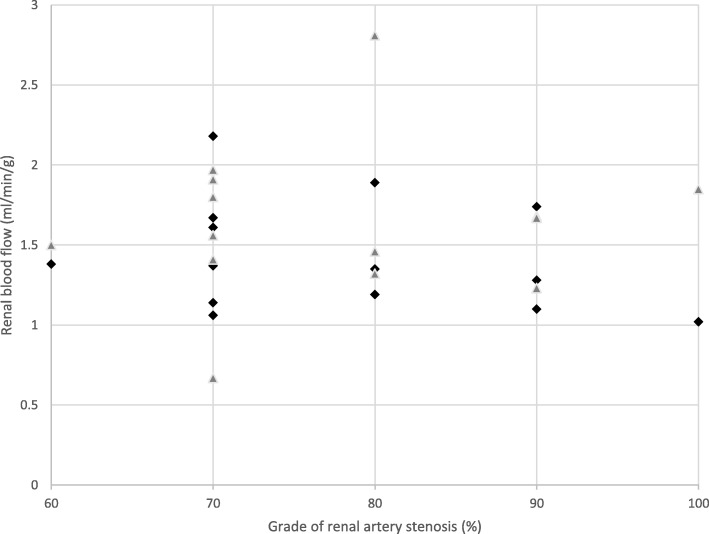
Grade of renal artery stenosis vs renal blood flow in bilateral RAS. ♦Grade of renal artery stenosis vs basal flow in bilateral RAS (*r* = 0.2, *p* = 0.4).  Grade of renal artery stenosis vs stress flow in bilateral RAS (*r* = 0.04, *p* = 0.9)

In the stenosed kidneys of unilateral stenosis, patients both basal and stress flow correlated negatively and statistically significant with stenosis grade (*r* = − 0.8, *p* = 0.04; *r* = − 0.8, *p* = 0.02, respectively) (Fig. 1). In bilateral stenosis patients, the degree of stenosis did not correlate with basal flow (*r* = 0.23, *p* = 0.40) or stress flow (*r* = 0.04; *p* = 0.89) (Fig. 2). We did calculate also Spearman correlation as sensitivity analysis. None of the conclusions changed. Flow values are reported in Table 2.

### Renal blood flow in diabetics and non-diabetics

In all the stenosed kidneys of non-diabetics, stenosis grade correlated statistically significant with basal flow and stress flow (*r* = − 0.7, *p* = 0.03; *r* = − 0.8, *p* = 0.007). In all the stenosed kidneys of diabetics, stenosis grade did not correlate with basal flow (*r* = − 0.3, *p* = 0.4) or stress flow (*r* = 0.01, *p* = 0.96). Flow values are reported in Table 2.

### Effect of 1 mg of enalapril on blood pressure

In the patients with bilateral artery, stenosis enalapril induced statistically significant decrease in MAP (predilatation MAP before and after enalapril 131 +/− 15 and 120 +/− 14 mmHg, respectively, *p* = 0.04). In unilateral RAS patients, MAP was 115 +/− 11 mmHg before enalapril and 117 mmHg after enalapril (*p* = 0.8). There was a statistically significant difference between MAP values of bilateral and unilateral RAS patients before enalapril (*p* = 0.03). In the healthy controls, MAP was 96 +/− 6 mmHg before and 97 +/− 7 mmHg after enalapril (*p* = 0.21).

Results of each patient separately are reported in Table 3.

**Table 3:** Basal and stress flow values, RFR, MAP, GFR, and grade of renal artery stenosis of each ARVD patient separately

No	Age/gender	RAS type	Grade of RAS (angiography) %	Basal flow pre (ml/min/g)	Stress flow pre (ml/min/g)	RFR pre (%)	MAP pre (mmHg)	MAP post (mmHg)	GFR pre (ml/min)	GFR post (ml/min)	DM
1	79/female	uni dx	70	1.4	1.2	− 10	132	123	38	38	–
		sin	0	2.2	2.0	− 10					
2	59/female	uni dx	60	2.8	2.5	− 9	122	108	78	87	–
		sin	0	2.7	2.7	2					
3	62/female	uni dx	0	1.0	1.7	68	104	101	63	83	+
		sin	90	0.8	0.8	0					
4	73/male	uni dx	60	1.7	2.6	50	110	125	105	113	–
		sin	40	1.9	2.7	40					
5	62/male	uni dx	0	0.9	1.1	27	129	136	72	82	–
		sin	90	1.0	1.2	25					
6	72/female	uni dx	90	1.1	1.4	20	113	125	39	39	–
		sin	45	2.3	2.5	8					
7	54/male	uni dx	0	1.5	1.7	11	106	129	68	61	–
		sin	60	1.5	1.9	26					
8	66/male	uni dx	70	–	–	–	107	122	26	26	+
		sin	0	–	–	–					
9	62/male	bi dx	80	1.9	2.8	49	126	113	31	26	+
		sin	100	1.0	1.9	81					
10	85/female	bi dx	60	1.4	1.5	9	138	140	56	61	+
		sin	80	1.2	1.5	23					
11	60/male	bi dx	70	1.1	0.7	− 41	141	116	51	69	+
		sin	70	2.2	1.4	− 35					
12	78/female	bi dx	90	1.7	1.7	− 3	117	123	77	63	+
		sin	80	1.4	1.3	− 2					
13	61/female	bi dx	90	1.3	1.2	−4	157	160	68	73	–
		sin	100	–	–	–					
14	71/female	bi dx	70	1.6	1.8	12	130	117	79	68	–
		sin	70	1.7	1.9	14					
15	85/female	bi dx	30	–	–	–	141	138	45	43	+
		sin	70	1.1	2.1	98					
16	52/female	bi dx	90	1.1	1.7	60	114	111	35	58	+
		sin	100	–	–	–					
17	84/male	bi dx	70	1.4	2.0	44	114	91	22	21	+
		sin	70	1.2	1.9	52					

*RAS* renal artery stenosis, *pre* predilatation, *MAP* mean arterial pressure, *post* postdilatation

## Discussion

In the present study, enalapril-stimulated RFR values were measured for the first time with [^15^O]H_2_O PET technique in healthy subjects and ARVD patients. RFR in the healthy was 22%. In unilateral RAS patients, RFR of the stenosed and contralateral kidneys were 15 and 21%, respectively. In the bilateral RAS kidneys, RFR was 27%. In all the stenosed kidneys of diabetics, RFR was 24% and of non-diabetics 14%. There was no difference of statistical significance between any of these values. Renovascular response to ACE inhibitor was different in unilateral and bilateral RAS.

RFR has been measured using different techniques almost all being invasive or non-quantitative. Doppler method is non-invasive, although not quantitative, as it is based on blood flow velocity and arterial diameter. PAH (p-aminohippuric)-clearance measures ERPF (effective renal plasma flow), but it gives only two-kidney perfusion if invasive techniques are not used. MRI (magnetic resonance imaging) technique has been used to measure renal blood flow, but there are only few studies of reliability and sensitivity of MRI so far [18].

PET provides a non-invasive and quantitative method to assess regional single-kidney perfusion values. However, only one study in which PET method was used has been published. In that study, RFR was assessed in hypertensive chronic kidney disease patients with quinaprilat. RFR was 26% which is in line with our study [14].

RFR values seem to depend on the imaging method as well as on the vasoactive medicine used. ACE-inhibitors have been used as vasodilators in many PAH, renography, and Xenon studies. In healthy normotensive men, ACE inhibitor-induced RFR has varied between 6 and 38% [19–21]. The RFR of the healthy subjects in our study is within these limits.

Despite of the same patient group, the same imaging technique, and the vasoactive substance, there still seems to be a high variability in RFR values. According to the literature, this phenomenon could be explained by salt consumption as well as by patient’s salt sensitivity [21–23]. The different activity of local renin system partly explains the variation of hemodynamic responses to RAA (renin-angiotensin-aldosterone)-inhibition [19] based on genetic polymorphism [24]. We did not use salt restriction in our study which may have effected on our results.

The data of the effect of ACE inhibitors on renal perfusion in renal artery stenosis is mainly based on animal studies or on renographic human studies [15, 16, 25–27]. In our study, renal perfusion increased after ACE inhibitor by 21% in the contralateral kidneys and by 15% in the stenosed kidneys of unilateral RAS. There is a human study with almost similar results with 99mTc-DTPA renography in unilateral RAS patients [16].

RFR of patients with bilateral RAS in our study was 27% which corresponds to captopril-induced 99mTc-DTPA renography measured RFR value of 24% in bilateral RAS patients [16]. In solitary kidney RAS, which mechanically resembles bilateral RAS, there are results which point to the same direction [25, 28, 29].

Basal and stress flow correlated negatively and statistically significantly with stenosis grade in unilateral RAS patients. In bilateral RAS patients, this connection between stenosis and flow disappeared and RFR tended even to increase with worsening stenosis grade. This can be explained by hemodynamic differences between unilateral and bilateral RAS. Diabetes is probably also an important explanation for this phenomenon because there were more diabetics in bilateral than in unilateral RAS patients. Diabetes has earlier shown to induce exaggerated responses to blockage of RAA system [30]. Likewise, in our study, RFR of the diabetics (24%) was higher than that of non-diabetics (14%).

In bilateral RAS, MAP decreased statistically significantly but no change of MAP was seen in unilateral RAS patients after ACE inhibition. This probably relates partly to differences in sodium intake and different activation of RAA system. Furthermore, in bilateral RAS, blood pressure and renal perfusion are more dependent on angiotensin II. In healthy subjects, there was no change of blood pressure either. However, the blood pressure change after ACE inhibition in healthy people is known to be smaller than in hypertensives especially when sodium depletion is used [21].

Dispersion of RFR values was high, which is a well-known phenomenon in renal [^15^O]H_2_O PET studies [31]. Juillard et al. [14] reported a renal blood flow increase of 26% with SEM of 7.2% which corresponds to SD of 20.4%. Furthermore, they fixed k1/k2 (pWater) to one in their studies that diminish dispersion.

Our study has certain limitations. The number of patients was relatively small, and there was unequal distribution of diabetics. Patients were not on salt restriction diet. Furthermore, we did not have any computed tomography associated with PET camera to differentiate the cortex and medulla of the kidney. Hence, cortical ROIs may have included an unknown admixture of medullary flow due to spatial resolution and partial-volume effect. We did not take any blood tests reflecting the function of RAA system.

## Conclusions

Our study is the first to report RFR of healthy people and RAS patients based on [^15^O]H_2_O PET. PET provides a non-invasive and quantitative method without contrast agent to evaluate renal blood flow even if high dispersion of values is challenging. More [^15^O]H_2_O PET-based studies of renal perfusion are under way and needed for validation of the technique.
